# Cervical cytology and associated factors among tribal women of Karnataka, India

**DOI:** 10.1371/journal.pone.0248963

**Published:** 2021-03-19

**Authors:** Supriti Ghosh, Sanjay M. Pattanshetty, Sneha D. Mallya, Deeksha Pandey, Vasudeva Guddattu, Veena G. Kamath, Shama Prasada Kabekkodu, Kapaettu Satyamoorthy, Ranjitha S. Shetty

**Affiliations:** 1 Department of Cell and Molecular Biology, Manipal School of Life Sciences, Manipal Academy of Higher Education, Manipal, Karnataka, India; 2 Department of Health Policy, Prasanna School of Public Health, Manipal Academy of Higher Education, Manipal, Karnataka, India; 3 Department of Community Medicine, Kasturba Medical College, Manipal Academy of Higher Education, Manipal, Karnataka, India; 4 Department of Obstetrics and Gynaecology, Kasturba Medical College, Manipal Academy of Higher Education, Manipal, Karnataka, India; 5 Department of Data Science, Prasanna School of Public Health, Manipal Academy of Higher Education, Manipal, Karnataka, India; 6 Centre for Indigenous Population, Kasturba Medical College, Manipal Academy of Higher Education, Manipal, Karnataka, India; Bharathidasan University, INDIA

## Abstract

**Background:**

Reproductive well-being is a crucial element of women’s health. Due to the asymptomatic nature of gynaecological morbidities, women rarely seek medical advice in the initial period leading to delayed diagnosis and poor prognosis of subsequent disease. The present study aimed to explore the cervical cytology and its associated risk factors among women from tribal communities of the southern part of coastal Karnataka, India.

**Methods:**

Papanicolaou (Pap) smear test was performed among 1140 women from three tribal populations, to detect cervical lesions, infections and reactive changes. A semi-structured questionnaire was administered to collect data on socio-demographic and reproductive characteristics of the study population.

**Results:**

The most predominant gynaecological complaint among the participants was severe lower back ache (77.6%), followed by white discharge per vagina (29.0%) and menstrual irregularities (25.9%). Of the 1140 women screened, 12.4% showed cervical microbial infections, 23.6% were reported to have reactive changes, and 0.2% had epithelial cell abnormalities in the cervix. Cervical microbial infections were found to be associated with younger age group, low socio-economic status and younger age at sexual debut.

**Conclusion:**

Most of the symptoms suggestive of gynaecological morbidities reported in this study are preventable or treatable. Strengthening ongoing cervical cancer screening programme and implementation of health education programmes among tribal population would be the right policy approach to prevent, detect and treat these symptoms at an early stage and to achieve acceptable health outcomes among tribal women.

## Introduction

Reproductive health is a crucial element of women’s health and well-being. Reproductive morbidities are the leading cause of disease burden among women in the reproductive age. In the low- and middle- income countries, five of every ten disability-adjusted life years (DALYs) among women aged 15–45 years are caused by reproductive ill-health [1]. According to the World Health Organization (WHO), reproductive morbidity is defined as “any morbidity or dysfunction of the reproductive tract, or any morbidity as a consequence of reproductive behaviour including pregnancy, abortion, childbirth and sexual behaviour”. It can be categorised as obstetric, gynaecological and contraceptive morbidity [2]. Gynaecological morbidities are the conditions of the women’s reproductive system including reproductive tract infections (RTIs), uterine prolapse, cervical cell anomalies, and infertility along with systemic conditions such as urinary tract infections (UTIs), high blood pressure, anaemia, obesity and syphilis but not associated with pregnancies [3].

In the resource-poor settings, socio-economic determinants such as low literacy rate, poverty, gender inequality, social stigma, lack of awareness, poor accessibility to healthcare services and limited decision-making power contribute to poor utilisation of reproductive health services, due to which most RTIs remain undiagnosed and untreated [4]. In addition, poor nutrition, poor menstrual hygiene, early marriage, prevailing sexually transmitted infections (STIs) and human immunodeficiency virus (HIV) infection lead to higher rates of gynaecological morbidity and mortality [5, 6]. Moreover, health is also adversely affected as women from low socio-economic background do not seek treatment in spite of perceiving most of their symptoms to be related to reproductive health issues [7].

Tribes account for about 8.6% of the total Indian population, with healthcare being a major concern in the isolated tribal areas [8]. A study conducted among the tribal population from another Indian state revealed high rates of female morbidity and mortality due to poor health-seeking behaviour for problems such as STDs, RTIs, menstrual disorders and unwanted pregnancies [9]. Kambo *et al*. reported the overall prevalence of at least one gynaecological complaint among women to be 24.4% and this percentage varied greatly (3.5% to 58.9%) with varying geographical locations across 14 Indian states (23 study districts) [10]. We attempted to study the cervical cytology and its associated factors among women from tribal communities residing in the southern coastal region of Karnataka, India.

## Materials and methods

### Ethical statement

The study was approved by State Tribal Welfare Department (STWD), Karnataka, Integrated Tribal Development Project (ITDP), District Health Authority, and Institutional Ethics Committee, Kasturba Hospital, Kasturba Medical College, Manipal (Registration No.: ECR/146/Inst/KA/2013, project approval no.: IEC181/2013). An informed written consent was obtained from the participants prior to data collection.

### Study setting and data collection

Over 40,000 individuals belonging to three tribal communities viz., Koraga, Marathi Naika and Malekudiya reside in the Udupi district of southern coastal Karnataka, India (Coordinates: 13.5401°N, 74.8741°E). A community-based cross-sectional survey was conducted among women from these three tribal communities from July 2014 to June 2017. The present study forms a part of a larger project wherein the baseline prevalence of viral infections of the cervix were explored. The sample size was calculated to be 1102, considering the prevalence of human papillomavirus (HPV) infection to be 16.9%, for a 15% precision at 95% confidence interval and 20% non-response rate [11, 12]. A total of 1140 women were recruited into the study.

Married tribal women in the age group of 20 to 65 years were included in the study. Women who were pregnant/ lactating at the time of study, those who had undergone hysterectomy or those who were previously diagnosed with cervical lesions and those with uterine prolapse were excluded from the study.

The eligible women from tribal communities were identified by a house-to-house survey. The socio-demographic, reproductive characteristics, along with information on symptoms suggestive of gynaecological morbidities among these women were collected by an interviewer using a semi-structured questionnaire (S1 and S2 Appendices). The detailed study design and data collection protocol have been described previously [12].

### Sample collection and cervical cytology

After seeking informed consent from the participants, cervical cells from the squamo-columnar junction were obtained by the doctor or a trained nurse using bivalve Cusco’s speculum and sterile Ayer’s spatula. A thin Papanicolaou (Pap) smear was prepared on a sterile glass slide, and the cells were fixed with 95% ethyl alcohol and air dried. The smears were analysed and reported according to the Bethesda 2001 system [13].

### Data analysis

Data was analysed using IBM SPSS version 26. The results are presented as frequencies and percentages. Prevalence of microbial infections was compared with various exposure variables using chi-square test. Poisson’s regression was used to find unadjusted and adjusted prevalence ratio with 95% confidence interval (CI) of microbial infections across the variables. For multivariable Poisson’s regression, variables were selected using backward elimination procedure. P-value >0.2 was used as an exit criteria for backward elimination procedure. In the last step, variables that were clinically relevant were included in addition to those that got selected from backward elimination procedure in multivariable Poisson’s regression analysis. A p-value <0.05 was considered statistically significant.

## Results

A total of 1140 women from Koraga, Malekudiya and Marathi Naika tribal communities in the age range of 20 to 65 years were identified and recruited into the study. About half of the study participants were in the age group of 31–45 years. The mean age at marriage was 21 (±3.8) years and the mean age at first pregnancy was 22.5 (±3.4) years. The mean difference between age at menarche and age at sexual debut was found to be 7.8 (±3.9) years. Almost two-thirds (61.1%) of the participants were from low socio-economic status and majority (85%) reported use of home-made sanitary napkins. Socio-demographic and reproductive characteristics of the participants are shown in Table 1.

**Table 1:** Socio-demographic and reproductive characteristics of the study participants (n = 1140).

Characteristic	No. (%)
**Age**	
≤30 years	259 (22.7)
31–45 years	572 (50.2)
>46 years	309 (27.1)
**Education level**	
Nil	392 (34.4)
≤7 years	519 (45.5)
>7 years	229 (20.1)
**Socio-economic status**	
Low	697 (61.1)
Medium	443 (38.9)
**Age at menarche**	
≤13 years	785 (68.9)
>13 years	355 (31.1)
**Age at first sexual intercourse**	
≤18 years	318 (27.9)
19–24 years	595 (52.2)
>24 years	22 (19.9)
**Age at first pregnancy**^a^	
≤22 years	633 (55.5)
>22 years	443 (38.9)
**Type of sanitary napkin used**	
Home-made	981 (86.1)
Disposable	159 (13.9)

^**a**^Sixty four (5.6%) of study participants were nulliparous.

Almost all (99.4%) the study participants reported to have at least one gynaecological complaint at the time of the survey, as shown in Table 2. Majority of the participants had three or more gynaecological morbidities (74.6%) followed by two morbidities (22.1%). Over 75% of the participants complained of severe lower-back ache, while white discharge per vagina was reported by 29% of the women. Menstrual problems were reported by 25.9% of the participants. Other gynaecological complaints including post-coital bleeding, dyspareunia and history of genital lesions were reported in less than one percent of the study participants.

**Table 2:** Distribution of gynaecological complaints among the study participants (n = 1140).

Gynaecological complaints	No. (%)
Severe lower-back ache	885 (77.6)
White discharge per vagina	331 (29.0)
Menstrual problems	
Dysmenorrhea	201 (17.6)
Irregular menstrual cycle	94 (8.2)
History of genital lesion	10 (0.9)
Dyspareunia	8 (0.7)
Post-coital bleeding	2 (0.2)
Number of gynaecological complaints	
Three or more	850 (74.6)
Two	252 (22.1)
One	31 (2.7)
None	7 (0.6)

### Exfoliative cervical cytology reports of the study population

Of the 1140 women screened for cervical lesions, 1138 (99.8%) were negative for intraepithelial lesions and malignancies (NILM). However, 141 (12.4%) women had cervical microbial infections, and two (0.2%) women were detected with abnormal epithelial cytology; one being high-grade squamous intraepithelial lesion (HSIL) and the other had atypical squamous cell of undetermined significance (ASCUS).

Among the women who were screened, candidiasis was the most common infection (6.1%) followed by coccobacilli (2.7%) and *Trichomonas vaginalis* infection (0.7%). In addition, 174 (15.3%) women had inflammatory smears, and 95 (8.3%) had atrophic smears. The distribution of cervical cytology reports of the study participants has been summarised in Table 3.

**Table 3:** Cervical cytology reports of the study participants (n = 1140).

Cytology report	No. (%)
NILM	1138 (99.8)
Epithelial cell abnormality	
ASCUS	1 (0.1)
HSIL	1 (0.1)
Microbial infections	
Candidiasis	69 (6.1)
Coccobacilli	31 (2.7)
Bacterial vaginosis	29 (2.5)
*Trichomonas vaginalis*	8 (0.7)
Altered vaginal flora	4 (0.4)
Reactive changes	
Inflammation	174 (15.3)
Atrophy	95 (8.3)
Unsatisfactory/ Inadequate smear	26 (2.3)

NILM: Negative for intraepithelial lesions or malignancies, ASCUS: Atypical squamous cell of undetermined significance; HSIL: High-grade intraepithelial lesions.

Table 4 shows the correlation of cytology findings and symptoms of gynaecologic morbidities. Of the women who had microbial infection and inflammation, the majority reported to have lower back ache (over 75%). Both the women with epithelial cell abnormality also claimed to have severe lower back ache. Pap smear test could not be performed for six (0.5%) participants with uterine prolapse.

**Table 4:** Cervical cytology findings in relation to symptoms suggestive of gynaecological morbidities among the study population.

Symptoms	NILM (n = 710)	ASCUS (n = 1)	HSIL (n = 1)	Microbial infection (n = 141)	Inflammation (n = 174)	Atrophy (n = 95)
	No. (%)	No. (%)	No. (%)	No. (%)	No. (%)	No. (%)
Irregular menstrual cycle (n = 94)	65 (9.2)	0	0	11 (7.8)	15 (8.6)	3 (3.2)
Dysmenorrhea (n = 201)	135 (19.0)	0	0	27 (19.1)	35 (20.1)	1 (1.1)
White discharge per vagina (n = 331)	234 (33.0)	0	0	45 (31.9)	46 (26.4)	4 (4.2)
Post-coital bleeding (n = 2)	2 (0.3)	0	0	0	0	0
Severe lower-back ache (n = 885)	551 (77.6)	1 (100.0)	1 (100.0)	112 (79.4)	134 (77.0)	83 (87.4)
Genital lesions (n = 10)	6 (0.9)	0	0	2 (1.4)	1 (0.6)	0
Dyspareunia (n = 8)	7 (1.0)	0	0	1 (0.7)	0	0

### Socio-demographic and reproductive characteristics associated with cytology findings

The association of cervical microbial infections with the socio-demographic and reproductive characteristics of the study population have been summarised in Table 5. Multivariable analysis was performed by Poisson’s regression, mutually adjusted for age, socio-economic status, difference between age at menarche and age at first sexual intercourse, age at first pregnancy, type of sanitary napkin used by the participants and presence of any gynaecological complaints. Regression analysis revealed that the prevalence of cervical microbial infections was 2.19 times (95% CI: 1.23, 3.93) higher among women of younger age, i.e., ≤30 years and 2.41 times (95% CI: 1.45, 4.01) higher among those aged between 31 and 45 years. Likewise, these infections were found to be 1.65 times (95% CI: 1.11, 2.47) higher among participants with low socio-economic status and 1.74 times (95% CI: 1.06, 2.85) higher among women who had a lesser difference between age at menarche and age at first sexual intercourse.

**Table 5:** Correlation of cervical infections with the socio-demographic and reproductive characteristics of the study population.

Characteristics	Cervical Microbial Infections		Unadjusted Prevalence Ratio (95% CI)	Adjusted Prevalence Ratio (95% CI)	p-Value
	Absent	Present			
	(n = 998)	(n = 141)			
	No. (%)	No. (%)			
**Age**					
≤30 years (n = 259)	219 (84.6)	40 (15.4)	2.51 (1.49, 4.23)	2.19 (1.23, 3.93)	0.008
31–45 years (n = 571)	489 (85.6)	82 (14.4)	2.34 (1.45, 3.77)	2.41 (1.45, 4.01)	0.001
>46 years (n = 309)	290 (93.9)	19 (6.1)	1.00	1.00	
**Marital status**					
Married (n = 1017)	888 (87.3)	129 (12.7)	1.29 (0.74, 2.26)	-	-
Widowed/Separated (n = 122)	110 (90.2)	12 (9.8)	1.00		
**Education level**					
Upto primary (n = 485)	568 (86.9)	86 (13.1)	1.16 (0.84, 1.59)	-	-
Middle school and above (n = 654)	430 (88.7)	55 (11.3)	1.00		
**Employment status**					
Employed (n = 507)	438 (86.4)	69 (13.6)	1.00	-	-
Home-maker (n = 632)	560 (88.6)	72 (11.4)	0.84 (0.61, 1.14)		
**Tribe**					
Koraga and Malekudiya (n = 450)	384 (85.3)	66 (14.7)	1.35 (0.99, 1.83)	-	-
Marathi Naika (n = 689)	614 (89.1)	75 (10.9)	1.00		
**Socio-economic status**					
Low (n = 697)	600 (86.1)	97 (13.9)	1.40 (0.99, 1.96)	1.65 (1.11, 2.47)	0.01
Medium (n = 442)	398 (90.0)	44 (10.0)	1.00	1.00	
**Difference between age at menarche and FSI**^a^					
≤3 years (n = 118)	97 (82.2)	21 (17.8)	1.51 (0.99, 2.31)	1.74 (1.06, 2.85)	0.03
>4 years (n = 1021)	901 (88.2)	120 (11.8)	1.00	1.00	
**Attained menopause**					
Yes (n = 319)	298 (93.4)	21 (6.6)	1.00	-	-
No (n = 820)	700 (85.4)	120 (14.6)	2.22 (1.42, 3.47)		
**Age at first pregnancy**					
≤22 years (n = 632)	559 (88.4)	73 (11.6)	0.88 (0.64, 1.22)	0.79 (0.54, 1.15)	0.21
>22 years (n = 443)	385 (86.9)	58 (13.1)	1.00	1.00	
**Parity**					
0–2 (n = 674)	582 (86.4)	92 (13.6)	1.00	-	-
>2 (n = 465)	416 (89.5)	49 (10.5)	0.77 (0.56, 1.07)		
**Types of sanitary napkin used**					
Home-made (n = 981)	863 (88.0)	118 (12.0)	0.82 (0.55, 1.25)	0.72 (0.44, 1.16)	0.18
Disposable (n = 158)	135 (85.4)	23 (14.6)	1.00	1.00	
**Presence of any gynaecological problems**^b^					
Yes (n = 1132)	992 (87.6)	140 (12.4)	0.99 (0.16, 6.23)	1.28 (0.78, 2.10)	0.33
No (n = 8)	7 (87.5)	1 (12.5)	1.00	1.00	

^a^FSI: First sexual intercourse;

^b^Includes menstrual cycle irregularity, history of dysmenorrhea, discharge per vagina, severe lower-back ache, post-coital bleeding, history of genital lesions, dyspareunia.

Cervical reactive changes including atrophy and inflammation did not show any statistically significant association with the risk factors.

The participant with HSIL was referred to the tertiary care hospital where colposcopy guided biopsy was performed for confirmation of the diagnosis, and the lesion was removed by Loop Electrosurgical Excision Procedure (LEEP). She and other participant with ASCUS were followed up every three months for a year. Women having bacterial vaginosis were treated with oral antibiotics, and those having vaginal candidiasis were managed with local antifungal treatments through the respective primary health centres.

## Discussion

In the present study, 1140 married women from the tribal population were screened for cervical premalignant/ malignant lesions, microbial infections and symptoms suggestive of gynaecological morbidities. The proportion of women having at least one gynaecological complaints in our study population was 99.4% which is much higher as compared to the findings of studies conducted in Maharashtra, India (75.7%), Brazil (78%), and Iran (79.4%) [14–16]. However, studies from rural regions of Kerala, Tamil Nadu and Uttarakhand states of India reported a lesser proportion of women having gynaecological complaints; ranging between 36.9% and 46.5% [17–19]. These discrepancies could be attributed to the differences in the type and profiles of the populations studied [20]. Zhang *et al*. reported that education status influenced the prevalence of gynaecological morbidities among women in China [21]. Similarly, a study by Abraham *et al*. reported that women with better education and easy accessibility to healthcare services were more likely to seek treatment for gynaecological symptoms at initial stages and hence, show reduced prevalence of gynaecological morbidities [17]. Our study participants constituted of a vulnerable population with low literacy level, low socio-economic status and distinct socio-cultural practices such as early age at marriage and pregnancy. This might also have contributed to the higher proportion of symptoms suggestive of gynaecological morbidities among these women.

In our study, majority of the women had three or more gynaecological complaints (74.6%). The higher number of gynaecological complaints among the study population may be attributed to the lack of awareness regarding gynaecological morbidities, poor accessibility to healthcare facilities in remote areas, embarrassment, stigma and fear towards pelvic examination, and above all the prevailing patriarchal family system in rural India, as indicated in prior studies [4, 7, 17, 20]. The predominant gynaecological complaint among the study population was lower back ache (77.6%) followed by white discharge per vagina (29.0%) and menstrual problems (25.8%). Gynaecological complaints reported in previous studies are similar to our study, though the proportion may vary [14, 17, 18, 20, 22, 23].

It was interesting to observe that in a population that has never been screened for cervical lesions, epithelial cell abnormalities such as ASCUS and HSIL were present in only 0.2% of the women. Previous studies have shown the prevalence of epithelial cell abnormalities of the cervix to be ranging from 1.7% to 16.0% [24–28]. The lower prevalence in our study can be attributed to younger age of our participants. Two third of the participants were having neither cervical cell abnormalities, nor any infections of the cervix and similar findings were reported from other studies across India with the prevalence ranging from 38% to 56% [23–25, 29].

In the present study, 6.1% of the women were found to have candidiasis which was slightly higher as compared with that of the findings from studies done by Arora *et al*. (4.2%) and Garcia *et al*. (4.5%) which included rural patients with RTIs [30, 31]. However, Choudhary *et al*. reported the prevalence to be as high as 12% in rural women suffering from RTIs and STIs. The proportion of coccobacillus infection and bacterial vaginosis among women in our study was 2.7% and 2.5%, respectively. Bhalla *et al*. reported the prevalence of bacterial vaginosis due to *Trichomonas vaginalis* and syphilis to be 28.8% in rural women which is much higher than that among our study population (5.9%) [32].

In our study, 15.3% of the women had inflammatory smears which is much lesser than that reported by Verma *et al*. (32.5%), Sachan *et al*. (42.7%), Kulkarni *et al*. (59.7%) and Atilgan *et al*. (74.5%) [24, 25, 33, 34]. The higher prevalence of inflammatory smears in these studies may be due to the fact that their population included women who were symptomatic and attending healthcare facility for the same, while ours was a population-based study in which screening was done among apparently healthy women. The proportion of atrophy was found to be 8.3% among the study population. A study by Kulkarni *et al*. reported the prevalence of atrophy to be lesser than our study findings (1.9%) while Babu *et al*. observed it to be 20.2% in their study [33, 35].

The present study reported an unsatisfactory/ inadequate smear rate of 2.3% whereas other studies reported it to be slightly higher with the rates being 4.8% and 6.4% [25, 36]. Unsatisfactory smears might be due to dryness of smear or scant cellularity among peri/post-menopausal women and could also be due to improper sample collection technique [25, 37].

In our study, women with cervical microbial infections and/ or inflammation were most commonly reported to have severe lower back ache, white discharge per vagina, dysmenorrhea and irregular menstrual cycle. Due to reasons such as these infections being generally asymptomatic or presenting with mild symptoms initially, hesitation among women to seek medical help or inaccessible health care system, women tend to neglect these symptoms [7, 17, 20].

Cervical microbial infections were found to be twice more prevalent among women of younger age (<45 years) and 1.6 times more prevalent among women with low socio-economic status. Contrary to our findings, Marrazzo *et al*. reported that the older women were at higher risk of cervicitis mediated by inflammation [38]. The positive association of presence of infections with young age could be attributed to the sexual activity among the younger women, which makes them vulnerable to various sexually transmitted microbial infections. Marconi *et al*. and Allsworth *et al*. reported an inverse relationship between income and presence of bacterial vaginosis [39, 40]. These studies are concordant to our data where low socio-economic status was found to be a risk factor for cervical infections. Deprivation has been linked with poor hygiene and non-responsiveness to regular health check-ups which can lead to accumulation and acquisition of newer infections [41]. Further, microbial infections were about 1.7 times more prevalent among women with less difference between age at menarche and age at first sexual intercourse. A study conducted by Ruiz et al. reported that a short interval between age at menarche and age at first sexual intercourse was associated with cervical abnormalities and high-grade lesions of the cervix [13].

This study, for the first time, reports the baseline prevalence of symptoms suggestive of gynaecological morbidities, cervical lesions, microbial infections and inflammation among women from a vulnerable community in this region. The data can be used for designing future studies and implementation of population-based interventions. As per the published literature it is noted that the prevalence of gynaecological morbidities has a wide range based on geography and population settings. This could be a limitation of the study, i.e., the data cannot be extrapolated to other populations from different resource setting. Further, due to the cross-sectional nature of the study, we cannot comment on the causative effect of the associated factors on cervical cytology.

## Conclusions

This study strengthens the existing evidence by presenting the information on prevalence and the factors associated with cervical cytology among the tribal population. To the best of authors’ knowledge, the present study is a first of its kind to provide such information on women belonging to vulnerable populations in this region. Most of the gynaecological symptoms documented in this study are preventable and treatable in nature. Hence, it is crucial to sensitise the tribal populations about the risk factors and prevention of gynaecological morbidities, and also strengthen the ongoing cervical cancer screening programmes, health promotion programmes, and early detection, referral and treatment strategies at the health system level. This would have policy relevance in prevention, detection and treatment of symptoms indicative of gynaecological morbidities at an early stage, leading to better follow-up and improvement in overall health status and well-being of tribal women.

## Supporting information

S1 Appendix Questionnaire in regional language (Kannada). (PDF)

S2 Appendix Questionnaire in English. (PDF)
