# Supplementary material for: Cervical cytology and associated factors among tribal women of Karnataka, India
Source: PLoS One. 2021 Mar 19;16(3):e0248963. doi: 10.1371/journal.pone.0248963 (PMC7978338; doi:10.1371/journal.pone.0248963)
Supplement: S1 Appendix — (PDF) [file pone.0248963.s001.pdf]

S1 Appendix. Questionnaire in regional language (Kannada)

ಪ್ರಶ್ನಾವಳಿ

ಮನೆ ಭೇಟಿ ಮಾಡಿದ ದಿನಾಂಕ: ____________________

1. ಸಾಮಾಜಿಕ ಮತ್ತು ಆರ್ಥಿಕ ವಿವರಗಳು:

1.1 ನೋಂದಣಿ ಸಂಖ್ಯೆ: ____________________

1.2 ವಯಸ್ಸು: ______________________ ವರ್ಷಗಳಲ್ಲಿ

1.3 ವೈವಾಹಿಕ ಜೀವನದ ಬಗ್ಗೆ ಮಾಹಿತಿ:

a. ವಿವಾಹಿತ / b. ಪತಿಯಿಂದ ಬೇರೆಯಾದವರು / c. ವಿಚ್ಛೇದಿತೆ / d. ವಿಧವೆ

1.4 ಶೈಕ್ಷ ಣಿಕ ವಿವರ: ಅಧ್ಯಯನ ದಲ್ಲಿ ಭಾಗವಹಿಸುವ ಮಹಿಳೆ __________

1. ಅನಕ್ಷರಸ್ಥ 2. ಪ್ರಾಥಮಿಕ 3. ಹಿರಿಯ ಪ್ರಾಥಮಿಕ/ ಮಾಧ್ಯಮಿಕ 4. ಪ್ರೌಢ ಶಾಲೆ 5. ಕಾಲೇಜು

1.5 ಉದ್ಯೋಗ: ಆಧ್ಯಯನ ದಲ್ಲಿ ಭಾಗವಹಿಸುವ ಮಹಿಳೆ _________

1.6 ಸಾಮಾಜಿಕ -ಆರ್ಥಿಕ ಸ್ಥಿತಿ: 1. ಕೆಳ ವರ್ಗ 2. ಮಧ್ಯಮ 3. ಉನ್ನತ

1.7 ಮದುವೆಯಾಗುವಾಗ ವಯಸ್ಸು:_________

1.8 ಪ್ರಥಮ ಬಾರಿ ಲೈಂಗಿಕ ಸಂಪರ್ಕ ಹೊಂದಿದ ವಯಸ್ಸು: ________

1.9 ಬುಡಕಟ್ಟು ಸಮುದಾಯದ ಹೆಸರು ______________________________

2. ಮುಟ್ಟಿನ ಬಗ್ಗೆ ಮಾಹಿತಿ:

2.1 ಮುಟ್ಟು ಆರಂಭವಾದ ವಯಸ್ಸು: _______

2.2 ಮುಟ್ಟಿನ ಅವಧಿ: ____________________ ಋತುಸ್ರಾವದ ದಿನಗಳು: _________________

2.3 ಮುಟ್ಟಿನ ಚಕ್ರ: ಕ್ರಮಬದ್ಧ ☐ ಕ್ರಮಬದ್ಧವಲ್ಲದ ☐ L.M.P ☐

2.4 ಮುಟ್ಟಿನ ದಿನಗಳಲ್ಲಿ ಕೆಳಹೊಟ್ಟೆ ನೋವು ಇರುತ್ತದೆಯೇ? ಹೌದು ☐ ಇಲ್ಲ ☐

2.5 ನಿಮಗೆ ಈಗ ಮುಟ್ಟು ನಿಂತಿದೆಯೇ? ಹೌದು ☐ ಇಲ್ಲ ☐

2.6 ಹೌದಾದರೆ, ಎಷ್ಟನೇ ವಯಸ್ಸಿನಲ್ಲಿ ಮುಟ್ಟು ನಿಂತಿತು? _________

3. ಪ್ರಸವದ ಬಗ್ಗೆ ಮಾಹಿತಿ:

3.1 ಮೊದಲು ಗರ್ಭಿಣಿಯಾದಾಗ ನಿಮ್ಮ ವಯಸ್ಸು ಎಷ್ಟು? ______________________

3.2 ಹಿಂದೆ ಗರ್ಭಿಣಿಯಾದದ್ದು (28 ವಾರಕ್ಕಿಂತ ಹೆಚ್ಚು) _______ ಜೀವಂತವಿರುವ ಒಟ್ಟು ಮಕ್ಕಳು ___________ ಗರ್ಭಪಾತ ___________ ಮಗು ಸತ್ತು ಹುಟ್ಟಿದ್ದು _________ ವೈದ್ಯಕೀಯ ಗರ್ಭಪಾತ ______________

4. ಸ್ತ್ರೀ ರೋಗಗಳ ಬಗ್ಗೆ ಮಾಹಿತಿ:

ಬಿಳಿ ಮುಟ್ಟು ☐ ಲೈಂಗಿಕ ಸಂಪರ್ಕ ಹೊಂದಿದ ನೊಂತರ ರಕ್ತಸ್ರಾವವಾಗುವುದು ☐

ತೀವ್ರವಾದ ಬೆನ್ನುನೋವು (ಕೆಳಭಾಗ) ☐

ಬೇರೆ ಏನಾದರೂ ಜನನಾಂಗದ ತೊಂದರೆಗಳು : ಹೌದು ☐ ಇಲ್ಲ ☐

ಹೌದಾದಲ್ಲಿ, ಎಷ್ಟು ಬಾರಿ ಆಗಿದೆ? _____________ ಪಡೆದುಕೊಂಡ ಚಿಕಿತ್ಸೆ _____________

ಏನೂ ತೊಂದರೆ ಇಲ್ಲ ☐

5. ವೈಯಕ್ತಿಕ ಮಾಹಿತಿ:

5.1 ನೀವು ಜನನಾಂಗವನ್ನು ಸಾಬೂನು ಮತ್ತು ನೀರಿನಿಂದ ದಿನಾಲೂ ತೊಳೆಯುತ್ತೀರಾ?

ಹೌದು ☐ ಇಲ್ಲ ☐

5.2 ನೀವು ಮುಟ್ಟಿನ ದಿನಗಳಲ್ಲಿ ಏನನ್ನು ಉಪಯೋಗಿಸುತ್ತೀರಿ? ಬಟ್ಟೆ ☐ ಸ್ಯಾನಿಟರಿ ಪ್ಯಾಡ್ ☐

5.3 ಅದನ್ನು ದಿನಕ್ಕೆ ಎಷ್ಟು ಬಾರಿ ಬದಲಾಯಿಸುತ್ತೀರಿ?___________________

ಶಿಬಿರದ ದಿನಾಂಕ: ______________________

6. ಪರೀಕ್ಷೆಗಳ ವಿವರ:

6.1 ಪ್ಯಾಪ್ ಸ್ಮಿಯರ್ ತಪಾಸಣೆಯ ವರದಿ: ____________________________________

6.2. ಮೇಲ್ದರ್ಜೆ ಅಸ್ಪತ್ರೆಗೆ ಹೆಚ್ಚಿನ ಪರೀಕ್ಷೆ ಮತ್ತು ಚಿಕಿತ್ಸೆಗಾಗಿ ಕಳುಹಿಸುವ ಅಗತ್ಯ ಇದೆಯೇ ?

ಹೌದು ☐ ಇಲ್ಲ ☐

ಕಳುಹಿಸಿದಲ್ಲಿ, ಪರೀಕ್ಷೆ ಮತ್ತು ಚಿಕಿತ್ಸೆಗಳ ವಿವರ: ______________________________
