# Supplementary material for: Cervical cytology and associated factors among tribal women of Karnataka, India
Source: PLoS One. 2021 Mar 19;16(3):e0248963. doi: 10.1371/journal.pone.0248963 (PMC7978338; doi:10.1371/journal.pone.0248963)
Supplement: S2 Appendix — (PDF) [file pone.0248963.s002.pdf]

S2 Appendix. Questionnaire in English

Date of house visit: __________________

1. Socio-demographic details:

1.1 Participant's ID: __________________

1.2 Age: _______ years

1.3 Marital status:

a. Married b. Separated c. Divorced d. Widowed

1.4 Education: Study subject: ____

1. Nil 2. Primary 3. Middle 4. High school 5. College

1.5 Occupation: Study subject: _____________

1.6 Socio-economic status: 1. Low 2. Medium 3. High

1.7 Age at marriage :

1.8 Age at first sexual intercourse:__________________

1.9 Tribal Community: ________________

2. Menstrual history

2.1 Age at menarche: ________________

2.2 Duration of cycle: _________________ Duration of bleeding:____________

2.3 Cycle: Regular / Irregular L.M.P: ________________

2.4 H/o Dysmenorrhea: Present / Absent

2.5 Attained menopause: Yes / No 2.6 Age at menopause: __________

3. Obstetric history

3.1 Age at first pregnancy:_____________

3.2 Obstetric score: Para (more than 28 weeks) ____ Living ____ Abortion____

Still birth ___ M T P ___

4. Gynecological complaints

White discharge ☐ Post coital bleeding ☐ Severe low back ache ☐

Any history of genital lesions? Yes ☐ No ☐

If Yes, details on frequency and treatment: ______________________________

No complaints ☐

5. Personal History

5.1 Do you clean introitus with soap and water daily? Yes ☐ No ☐

5.2 What type of sanitary napkins do you use during menstrual periods?

Home-made ☐ Disposable ☐

5.3 How frequently you change sanitary napkins in a day? ___________________

Date of camp: __________________

6. Investigation reports:

6.1 Pap smear report: ____

6.2 **Referral**

Referred to Hospital: Yes ☐ No ☐

If Yes, Treatment details: ____
